# Polystyrene Sulfonate Particles as Building Blocks for Nanofiltration Membranes

**DOI:** 10.3390/membranes12111138

**Published:** 2022-11-12

**Authors:** Philipp Jahn, Michael Zelner, Viatcheslav Freger, Mathias Ulbricht

**Affiliations:** 1 Institute of Technical Chemistry II and Center for Water and Environmental Research, University of Duisburg-Essen, 45117 Essen, Germany; 2 Wolfson Department of Chemical Engineering, Technion-Israel Institute of Technology, 3200003 Haifa, Israel

**Keywords:** nanofiltration, polyelectrolyte complex membrane, polystyrene sulfonate particles, charged mosaic membranes

## Abstract

Today the standard treatment for wastewater is secondary treatment. This procedure cannot remove salinity or some organic micropollutants from water. In the future, a tertiary cleaning step may be required. An attractive solution is membrane processes, especially nanofiltration (NF). However, currently available NF membranes strongly reject multivalent ions, mainly due to the dielectric effect. In this work, we present a new method for preparing NF membranes, which contain negatively and positively charged domains, obtained by the combination of two polyelectrolytes with opposite charge. The negatively charged polyelectrolyte is provided in the form of particles (polystyrene sulfonate (PSSA), d ~300 nm). As a positively charged polyelectrolyte, polyethyleneimine (PEI) is used. Both buildings blocks and glycerol diglycidyl ether as crosslinker for PEI are applied to an UF membrane support in a simple one-step coating process. The membrane charge (zeta potential) and salt rejection can be adjusted using the particle concentration in the coating solution/dispersion that determine the selective layer composition. The approach reported here leads to NF membranes with a selectivity that may be controlled by a different mechanism compared to state-of-the-art membranes.

## 1. Introduction

Nanofiltration (NF) is gaining increasing importance because it offers new possibilities for more effective water purification and it has also great potential for the recovery of valuable resources from water [1]. In many cases, a tailored selectivity, for instance, between different ions, is of large interest, but the permeance of the membrane should also be competitive. Most frequently used commercially available NF membranes are thin-film composite membranes, most of which are fabricated using the interfacial polymerization of polyamides as a separation layer [2]. One of the promising emerging alternatives is NF membranes with polyelectrolytes as building blocks for their separation layer, with the layer-by-layer (LBL) technology as one effective fabrication method [3,4]. The combination of polymers with complementary charged groups (polyelectrolytes) on a suited ultrafiltration (UF) membrane forms a selective thin film with controllable properties. To obtain such thin films, different LBL methods can be used, e.g., dip coating, spray coating, and spin coating [3,4,5]. The formation and structure of these films are strongly influenced by pH, ionic strength, and temperature. The LBL process in general is not limited to polymeric materials; for instance, it can be used to prepare layers from charged particles [6]. The most significant drawbacks of LBL-enabled processes are the cumbersome multi-step coating process and the fact that the polyelectrolyte-based membranes may exhibit a lack of stability to high ionic strengths and extreme pH values. Membranes prepared using the LBL method often exhibit very similar separation properties compared to simply charged NF membranes because the separation performance is often largely determined by the last applied layer [7]. LBL-prepared polyelectrolyte membranes are stable in organic solvents; therefore, they are suitable for solvent-resistant nanofiltration (SRNF) [8].

The use of a combination of polyelectrolytes of opposite charge in membrane fabrication, e.g., via the LBL process, results in polyelectrolyte complex (PEC) membranes. Polyelectrolyte membranes have been known for many decades and were first described by Meyer and Sievers in the 1930s [9,10]. The transport through these membranes is described by the Donnan Steric Pore Model with Dielectric Exclusion (DSPM-DE) theory [11]. Different contributions to selectivity can be discussed, based on three mechanisms, i.e., size exclusion, Donnan exclusion, and dielectric exclusion. The selectivity of different types of NF membranes is affected differently by individual contributions. Membranes whose selectivity is based to a large extent on dielectric exclusion are characterized by the fact that they often have a higher rejection of multivalent ions compared to monovalent ions. For example, the well-known polyamide membrane NF270 from DuPont exhibits a strong dependence on dielectric exclusion due to its dense structure, low dielectric constant, and limited swelling in water. By this membrane, both kinds of divalent ions, cations and anions, are more rejected than monovalent ions; e.g., single salt rejection of both MgCl_2_ (CaCl_2_) and Na_2_SO_4_ is higher than that of NaCl (MgCl_2_ (CaCl_2_) = Na_2_SO_4_ > NaCl), although the membrane has a negative surface charge [12]. This also results in a higher scaling tendency for typical scalants, such as hydroxylapatite (Ca_5_[OH(PO_4_)]_3_) or calcium sulfate (CaSO_4_). Conversely, when the selectivity of the membrane is strongly dependent on Donnan exclusion, which is the case when relatively loosely bound swellable polyelectrolytes are used to build the selective layer, the scaling tendency can decrease due to the depletion of one of the scaling forming species. An example of a negatively charged NF membrane, with high dependence on Donnan exclusion, was presented by Bernstein et al. [13]. This membrane was synthesized by grafting cross-linked poly(vinyl sulfonic acid) onto an UF membrane, leading to a strongly negatively charged selective layer of the NF membrane. This also led to a much higher rejection of negatively charged ions than positively charged ions (Na_2_SO_4_ > NaCl > CaCl_2_). These membranes showed a significantly lower scaling tendency compared to commercial polyamide (PA) membranes [14]. Previous work of Levchenko and Freger [12] demonstrated that cross-linked polyethyleneimine (PEI) generates a positively charged NF selective layer. This membrane was prepared by crosslinking of PEI on a suitable UF membrane support. Due to the strong positive charge, the single-salt rejection sequence was MgCl_2_ > NaCl > Na_2_SO_4_. In addition, this membrane also showed a significantly lower scaling tendency for phosphates and sulfates, based on the depletion of these species from the retentate.

The combination of loosely bound, swellable polyelectrolytes of different charge can lead to a charged mosaic (CM) membrane. The concept of CM membrane was developed by Sollner in 1932 [15]. The selective layer of CM membranes is characterized by differentially charged domains, and their separation mechanism is also strongly influenced by Donnan exclusion [16,17]. This leads to a depletion of both kinds of charged species of higher charge density via interaction with the complementary domains and results in a unique rejection pattern (NaCl > Na_2_SO_4_ ~ CaCl_2_). This could also be an advantage for scaling prevention, but it of interest as well for tertiary treatment of saline wastewater, where there is interest in removal of NaCl, but ions such as Ca^2+^, Mg^2+^, or HPO_4_^2−^ should remain in the treated water. CM membranes have been under development for a long time. A CM membrane was prepared, for example, via demixing of a charged and an uncharged polymer during membrane casting and subsequent functionalization of the uncharged polymer with oppositely charged groups [16]. However, no CM membrane with the competitive selectivity and permeability can yet be fabricated with a well-scalable method [3].

In a recent perspective article on new materials and approaches to membrane fabrication [18], the utilization of nano- and microparticles as building blocks for membranes was also emphasized as one promising route. Very much research is devoted to nanocomposite membranes with porous inorganic or organic/inorganic particles as part of the selective layer [19]. However, the focus of this work is on purely organic particles that can act as permeable domains in the selective layer. This approach is much less explored. Among the few examples in the literature are zwitterionic polymeric nanoparticles that have been integrated via interfacial polymerization into PA layers [20].

In this work, we present a new method for preparing NF membranes, which contain negatively and positively charged domains, obtained by the combination of two polyelectrolytes with opposite charge. The negatively charged polyelectrolyte is provided in the form of particles with a diameter of about 300 nm. Particles are synthesized by batch emulsion polymerization of 4-styrene sulfonic acid ethyl ester with divinylbenzene as a crosslinker monomer and subsequent conversion to polystyrene sulfonate (PSSA). As a positively charged polyelectrolyte, PEI is used to act as the matrix for incorporation/immobilization of the PSSA particles. Both buildings blocks and glycerol diglycidyl ether (GDE) as the cross-linker for PEI are applied to a UF membrane support in a simple one-step coating process, followed by thermal curing. The fraction of PSSA particles in the coating solution/dispersion was varied, and this yielded tunable composition and net charge of the selective layer, as shown by IR spectroscopy and zeta potential analyses. NF characterization revealed that the salt rejection could also be tuned from the typical behavior of a cationic membrane (without PSSA) to that of an anionic membrane (at a high PSSA content). For medium values of PSSA concentration used for the coating, it was possible to obtain net-charge-balanced NF membranes that had equal rejections of Na_2_SO_4_ and CaCl_2_ and lower rejection of NaCl. Hence, the feasibility of integration of polyanionic particles as building blocks in PEC NF membranes was demonstrated, but no CM behavior could be obtained.

## 2. Materials and Methods

### 2.1. Materials

Polyethersulfone (PES) flat sheet ultrafiltration membranes with a molecular weight cut-off (MWCO) of 30 kDa, provided by Sartorius (type: 14659, batch number: 2050123), were used as a support membrane. Divinylbenzene (DVB) from Fluka was used as a crosslinker monomer. The functional monomer styrene sulfonate sodium salt (SSA-Na), bromoethane (EtBr), potassium persulfate (KPS), polyethyleneimine (PEI), 270 kDa), crosslinker glycerol diglycidyl ether (GDE), and the surfactant sodium dodecyl sulfate (SDS) were purchased from Sigma Aldrich. The solvents acetonitrile and dichloromethane were obtained from VWR. The salts sodium chloride (NaCl), sodium sulfate (Na_2_SO_4_), calcium chloride (CaCl_2_), and sodium phosphate (Na_3_(PO_4_)_2_) were received from Fluka. Silica gel (for chromatography) with a particle size of 60–200 µm from Acros Organics was used for the purification of the monomer. All chemicals were used as received. Ultrapure water was provided by the water purification system Arium from Sartorius (Göttingen, Germany).

### 2.2. Preparation of Negatively Charged Polyelectrolyte Particles (Polystyrene Sulfonate)

**Figure 1:**
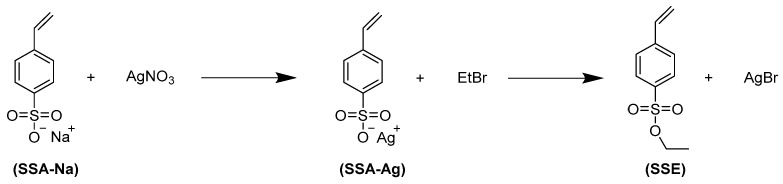
Reaction scheme for synthesis of styrene sulfonic acid ethyl ester.

The polyelectrolyte particle synthesis via emulsion polymerization of a hydrophobic precursor, the protected polystyrene sulfonic acid, and subsequent deprotection was based on the works of Tiwari and Walther [21] and Woeste et al. [22]. Since the monomer styrene sulfonic acid ethyl ester (SSE) was not readily available, it was synthesized (Figure 1). The silver method was used to convert the sodium salt of the monomer into the corresponding sulfonic acid ester [23].

**Figure 2:**
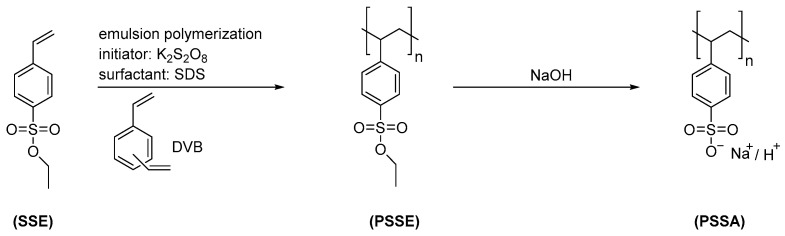
Reaction scheme for synthesis of the particles via emulsion polymerization of SSE and DVB initiated by KPS in an aqueous SDS solution, followed by saponification of the sulfonic acid ester (“deprotection” of ion exchange groups).

First, SSA-Na was dissolved in water, and silver nitrate in a molar ratio 1:1 was added as solid under cooling at 4 °C and protection against light. The precipitated grey solid was separated via suction filtration and washed several times with ice-cold water and diethyl ether. Then, the grey product was dissolved in acetonitrile and filtered again to remove impurities. For the second step, the double molar amount of EtBr relative to SSE-Ag was added, and the reaction was carried out for six hours at 70 °C. After cooldown, the solution was filtrated via suction filtration to remove the co-product silver bromide. The solvent was then removed by rotary distillation. Afterwards, the white residue was dissolved in dichloromethane, and the solution was purified by passing it through a column containing silica gel. Finally, the solvent was removed using rotary distillation. The final product was a slightly yellowish viscous liquid and was stored in a freezer at <−21 °C to prevent auto-polymerization. The purity of the product SSE was confirmed by ^1^H-NMR spectroscopy. The particles were prepared by emulsion polymerization of SSE with DVB as a crosslinker, using SDS as a surfactant and KPS as an initiator (Figure 2). A different reactor from Tiwari and Walter [20] was used, and some reaction conditions were adjusted. First, 200 mL of a solution of SDS in water with a concentration of 0.5 mmol/L was filled into the small lab-scale glass reactor with a mechanical stirrer (instead of using snap-on glass vials with magnetic stirring bar). After degassing of the SDS solution in a vacuum chamber at 200 mbar, the monomer mixture with 1 wt% SSE relative to continuous phase and 4 mol% DVB (relative to total monomer) was added. After heating to 70 °C, the mixture was stirred at 800 rpm for 30 min with a mechanical anchor stirrer. Then, KPS dissolved in a small amount of water was added to the reactor; the concentration of KPS in the mixture was 4 mmol/L. After a few seconds to minutes, the emulsion changed from turbid to a white dispersion. To ensure complete monomer conversion, the reaction was continued for 24 h. The mixture was then filtrated with an MN615 ¼ pleated filter paper (corresponding retention range > 4 µm) from Macherey-Nagel to remove big structures and thereafter filled in a dialysis bag with a nominal MWCO of 12 kDa and dialyzed against DI water. After reaching a conductivity of < 5 µS/cm in the dialysate, purification was considered complete. Then, the particles were freeze-dried (Martin Christ Alpha 1-4 100400 ISCEON, Osterode, Germany); a cotton wool-like solid was obtained. For deprotection, the particles were dispersed in 1 mol/L aqueous sodium hydroxide solution and heated to 110 °C under reflux for 12 h (Figure 2). These harsh conditions ensure a complete conversion of the sulfonic acid ester. The purification was carried out again by dialysis (MWCO 12 kDa) until a conductivity < 5 µS/cm was reached. Obtained particles were again freeze-dried.

### 2.3. Particle Characterization

#### 2.3.1. Calculation of Charged Group Density

The charged group density (CGD) was calculated following Equation (1),

(1) $$CGD=\frac{z}{x_{1}\cdot M_{1}+x_{2}\cdot M_{2}}$$

where z is the charge per repeat unit and $M_{1}$ is the molar mass of functional monomer,$\;M_{2}$ is the molar mass of crosslinker monomer, and $x_{1}$ and $x_{2}$ are the molar fractions of functional monomer and crosslinker monomer, respectively, in the copolymer.

#### 2.3.2. Zeta Potential and Particle Size

The particles were re-dispersed in ultrapure water at a concentration of 1 mg/mL. Dynamic light scattering (DLS) measurements were performed on a Zetasizer UltraPro from Malvern Panalytical (Worcestershire, UK) equipped with a DTS1070 flow cell. After determining particle size, the zeta potential was analyzed in the same cell. The PDI for an individual peak of the particle size distribution was calculated with Equation (2).

(2) $$PDI={\left(\frac{\sigma }{d}\right)}^{2}$$

where σ is the standard deviation and d is the mean particle size. For measuring the pH dependency, an automatic titration unit (MPT-2) was connected to the Zetasizer instrument. The measurements were performed with a pH increment of 0.5. The pH was adjusted by using HCl or NaOH, respectively.

#### 2.3.3. Scanning Electron Microscopy (SEM)

For SEM image acquisition, the particles were first dispersed in water. In parallel, a single crystalline silicon wafer was immersed in a 10 g/L solution of PEI (270 kDa, branched) in water and cleaned with water after 10 min. Subsequently, the wafer was dried with compressed air and then immersed in the particle dispersion for another 10 min, followed by rinsing with water. Due to the electrostatic interactions between the PEI on the wafer surface and the particles, single particles could be imaged. To ensure sufficient conductivity of the sample, the samples were sputtered with an Au/Pd layer. The image acquisition was performed with the instrument Apreo S LoVac from Thermo Fisher Scientific (Waltham, MA, USA).

### 2.4. Membrane Fabrication

The support membrane was cut into rectangular shape (130 mm × 210 mm). It was first washed with a mixture of water/ethanol (50:50) for two hours to remove soluble components. Then, the membrane was soaked in a solution of 50 g/L glycerol in ethanol for 24 h. Afterwards, it was mounted in a glass frame (120 mm × 200 mm) that allows it to cover the membrane with a solution. The coating solution/dispersion with the desired concentrations was prepared by adding PSSA particles to a solution of PEI in ethanol solution, followed by sonication for 20 min to ensure that the particles were also well dispersed. Finally, the crosslinker (GDE) was added, and the solution/dispersion was stirred for 20 min. In the meantime, the surface of the mounted membrane was washed with ethanol a few times to remove the excess of glycerol from the surface, followed by a quick drying of the surface with compressed air. Next, the modification solution was spread on the membrane surface, limited by the glass frame; the volume/area ratio was always ~0.28 mL/cm^2^. This value was chosen to ensure a complete coverage of the membrane surface with liquid. After 5 min, the liquid was discarded, and the wet membrane was transferred to an oven where is was kept in horizontal orientation at 60 °C for two hours to ensure a complete cross-linking of PEI by GDE.

### 2.5. Membrane Testing and Characterization

#### 2.5.1. Water Permeance and Single Salt Rejection

The performance of the membrane was determined in a laboratory dead-end nanofiltration set-up equipped with a stirrer. The feed container had a volume of 100 mL. The active membrane area was 9.62 cm^2^ and the stirring rate was set to 600 rpm. Each membrane sample was fully compacted by pure water filtration at 8 bar until constant flux was reached, before testing separation performance. Water permeance was calculated by Equation (3).

(3) $$P=\frac{V}{p\cdot t\cdot A}\;$$

where V is the filtered volume, p is the transmembrane pressure, t is the sampling time, and A is the active membrane area. The rejection of NaCl, Na_2_SO_4_, and CaCl_2_ was determined with single salt feed solutions containing 1 g/L of the individual salts in water. Conductivity was measured to determine salt concentrations. Rejection was calculated by Equation (4).

(4) $$R=\left(1-\frac{C_{P}}{C_{F}}\right)\cdot 100\%$$

where $C_{P}$ and $C_{F}$ are salt concentrations in initial feed and in collected permeate, respectively. The mixture of the three salts (0.25 g/L Na_2_SO_4_, 0,25 g/L CaCl_2_, and 0.5 g/L NaCl) at a total concentration of 1 g/L was also used. The cation and anion concentrations in mixed salt solution of feed and permeate were determined separately. The cations were analyzed via atomic absorption spectroscopy (AAS) using M-Series FS95 from Thermo Fisher Scientific (Waltham, MA, USA), and for the anions, an ion chromatograph from Metrohm (IC 883 with Autosampler; Herisau, Switzerland) was used. For all filtrations, a maximum of 20 mL of permeate was filtered through the membrane to avoid a too strong concentration of the feed (maximum concentration factor of 1.25).

Three samples for each membrane type have been tested and mean values and standard deviations are reported.

#### 2.5.2. Zeta Potential

Zeta potential of the membrane surface was determined by using a SurPASS1 electrokinetic analyzer from Anton Paar (Graz, Austria) equipped with an adjustable gap cell. The gap width was adjusted to 100 µm with a tolerance of 5 µm. The measurement was performed with 1 mmol/L KCl solution as electrolyte. At the beginning of each measurement, 550 mL of that KCl solution was added to a container, and the pH value was adjusted to a value of ~2.5. After 10 min of circulating the solution through the measurement cell, the measurement was started. During measurement, the pH value was automatically adjusted with an increment of 0.5 by using 0.1 mol/L KOH solutions. At every pH increment, a triple determination was performed. The zeta potential was calculated by using the Helmholtz–Smoluchowski Equation (5).

(5) $$\zeta =\frac{dU_{str}}{dp}\cdot \frac{\eta }{\epsilon \cdot \epsilon_{0}}\cdot {Κ}_{B}$$

where $\frac{dU_{str}}{dp}$ is the slope of the plot streaming potential vs. differential pressure, ${Κ}_{B}$ is electrolyte conductivity, η is electrolyte viscosity, ϵ is dielectric constant of electrolyte, and $\epsilon_{0}$ is permittivity of vacuum.

#### 2.5.3. ATR-IR Spectroscopy

The surface chemistry was characterized using FTIR spectroscopy in the attenuated total reflectance (ATR) mode (Bruker Alpha I). The membrane sample was measured at three different locations in the range 400–4000 cm^−1^.

## 3. Results and Discussion

### 3.1. Poly(Styrene Sulfonic Acid) Particles

PSSA particles were synthesized as described in Section 2.2. The mechanical stirring system was used instead of simple magnetic stirring to have more control over the stirring speed. The emulsion polymerization was performed using a surfactant (SDS) concentration (0.5 mmol/L), which was well below its critical micelle concentration (CMC ~8.2 mmol/L) because it is well-known that emulsion polymerization below the CMC also lead to well-defined particles [24,25]. Lower SDS concentrations produce fewer nuclei during nucleation and lead to growth of larger particles and vice versa [21]. The specific particles selected for this work had been obtained with a crosslinker content of 4 mol% DVB in the dispersed organic phase consisting of the monomer SSE (Figure 2). The resulting moderate cross-linking degree should on the one hand provide sufficient swelling in water to allow ion transport through the particles and on the other hand yield sufficient particle stability. Table 1 shows the most important properties of the obtained particles. The target size of 200 nm was approximately obtained for the protected version (207 ± 12 nm) of the particles by using 0.5 mmol/L SDS and 4 mol% DVB with 1 wt% SSE (compared to continuous phase). Furthermore, the sulfur content was determined by elemental analysis of the dried particles and used to calculate the actual density of functional or charged groups. The values were only slightly lower than the theoretical values of 4.8 mmol/g for protected and 4.9 mmol/g for deprotected particles, calculated by Equation (1) for complete incorporation of both monomers in the copolymer.

**Figure 3:**
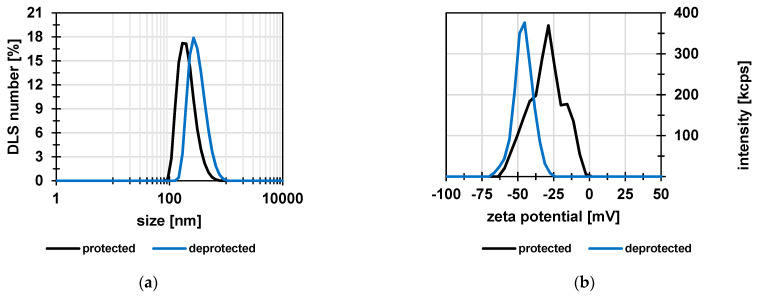
Comparison of protected and deprotected particles: (**a**) size; (**b**) zeta potential (kcps = kilo counts per seconds); data are shown for one of the three independent measurements (Table 1).

Average particle size of as-synthesized particles increased from 207 nm to 344 nm after complete hydrolysis of all ester groups to yield sulfonic acid groups; this indicated significant swelling of the particles in water (Figure 3a, Table 1). The swelling is driven by the hydration of the charged groups of the polymer and counteracted by the chemical crosslinking of the network. The PDI was low and did not change after deprotection. The size distribution could be described as practically monodisperse. Moreover, the introduction of the sulfonic acid groups shifted the zeta potential after deprotection of the particles to more negative values (Figure 3b). The fact that the protected particles also showed a negative zeta potential can be explained by incorporation of the surfactant SDS, with sulfate groups, on the particle’s surface.

**Figure 4:**
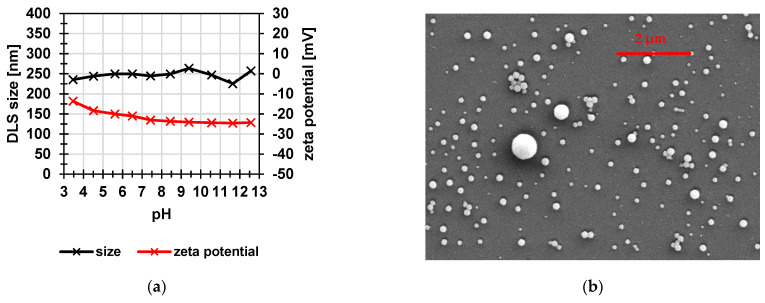
(**a**) Particle size and zeta potential as function of pH value; (**b**) SEM image of protected particles deposited on a PEI-coated silicon wafer. Measured particle size 133 ± 27 nm (*n* > 100).

To investigate the stability of the particles, their size and zeta potential in water were measured as function of pH value (Figure 4a). The size varied only slightly between 235 nm and 257 nm. The zeta potential decreased slightly in the acidic pH range. These results proved that the particles are negatively charged and that, consequently, their swelling degree did not change significantly over the entire pH range. Figure 4b shows an SEM image of the protected particles. The observed size was 133 ± 27 nm and therefore smaller than the values determined by DLS. This is due to the dry state of the particles, because the DLS method determines the hydrodynamic diameter, which is usually larger because of hydration effects.

### 3.2. Membrane Performance

**Figure 5:**
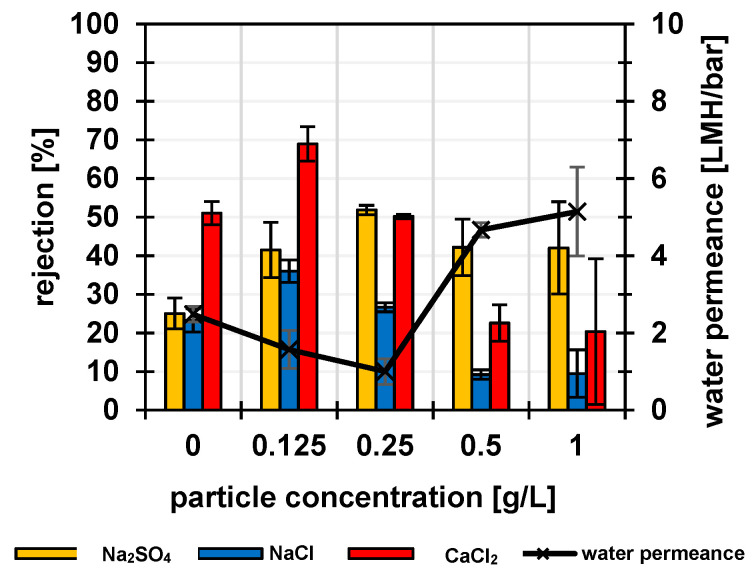
Influence of the variation of PSSA particle content in ethanol used for coating the porous PES support membrane onto the water permeance and rejection of single salts. The PEI and GDE contents were 0.5 g/L and 1 g/L, respectively.

As the cationic matrix for incorporation of the particles, branched PEI with a molar mass of 270 kDa was used. The particles were limited in their swelling in water by crosslinking during synthesis by polymerization (Section 3.1), whereas the matrix polymer PEI should be cross-linked by GDE. The coating of the porous PES support membranes was carried out with ethanol instead of water as solvent, because the crosslinker GDE is insoluble in water. To increase pore stability during drying, the membranes were impregnated, before coating, in a glycerol/ethanol mixture. Glycerol cannot evaporate at the given conditions and thus additionally stabilizes the pores. In the first series of experiments, the PEI and the crosslinker concentrations were kept constant at 0.5 g/L and 1 g/L, respectively, while the particle concentration was varied from 0 to 1 g/L; results are shown in Figure 5. The relatively small errors of the measurements for three samples of 9.62 cm^2^ from the same membrane batch indicate that the membrane fabrication is uniform on the cm length scale. The NF experiments with the single salt solutions were typically performed by first using the NaCl solution, followed by the Na_2_SO_4_ solution and then the CaCl_2_ solution; finally, the NaCl solution was filtered again. The NaCl rejection values in first and last filtrations were identical within the margin of error, indicating that the membranes were stable during the series of filtrations of different salt solutions. The reference membrane without addition of particles already showed an Na_2_SO_4_ rejection of 24%, a similar rejection for NaCl and the highest rejection for CaCl_2_. This can be explained by the positively charged barrier layer composed of cross-linked PEI. The amino groups were protonated and therefore positively charged and thus increased the rejection based on Donnan exclusion, especially for double-positively charged Ca^2+^ ions. The observed reduced water permeance and increased rejection for all single salts at low addition of PSSA particles (up to 0.125 g/L) indicated the promotion of crosslinking of the PEI-based barrier layer by the particles. Because of the small PSSA fraction, the effect of PEI on rejection still dominated, so that rejection of CaCl_2_ was still highest. With a particle concentration of 0.25 g/L in the coating solution/dispersion, the resulting membrane had approximately equal rejections (~50%) for CaCl_2_ and Na_2_SO_4_ and ~25% rejection for NaCl. Similar rejection of the salts with the two double-charged ions of opposite charge indicated a macroscopically neutral membrane barrier layer. For that kind of membrane, it had also been found that the rejection of the individual ions in a ternary salt mixture of the same total salt concentration was, within the range of error, identical to the data obtained for single salt feeds (Figure S1).

By increasing the particle content further, up to 1 g/L, the water permeance increased strongly and salt rejection decreased. However, the influence of the negatively charged particles on salt rejection became larger because the rejection of Na_2_SO_4_ was much higher than for the other salts. Hence, the particles dominated the properties of the selective layer. However, they seemed to interfere with the chemical cross-linking of the PEI. Instead, the proportion of ionic crosslinking, due to interactions between PEI (+) and particles (-), became larger. This formation of polyelectrolyte complexes, in combination with a lower degree of chemical crosslinking of the layer, could be considered a reason for higher water permeance and overall lower salt rejection.

In a second series of experiments, both PEI and crosslinker concentrations were kept constant at 1 g/L, and the particle concentration was varied from 0 to 1 g/L; results are shown in Figure S2. Because of the higher PEI concentration, the salt rejection of the reference membrane (0 g/L PSSA) was much higher than in the first series and similar to other nanofiltration membranes with the cross-linked PEI layer reported in the literature [12]. The effects of the PSSA particles were similar to the first series, but because of the higher rejection values, the trends were less clear. Therefore, the further analysis is focused on composite membranes from the first series. Overall, the permeance of the obtained membranes was rather low compared to other NF membranes, with PEI as part of the selective layer (e.g., [12,26]). The main reason is likely that the PES UF membrane that had been used as support had not been developed for this purpose and that its structure had not been fully protected against pore collapse during the thermal curing step.

**Figure 6:**
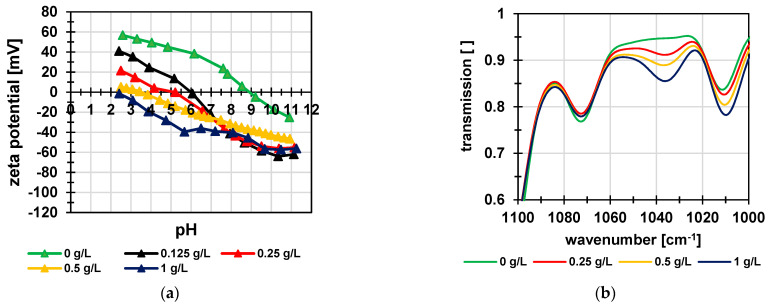
(**a**) Zeta potential as function of pH value; (**b**) IR spectra; membranes coated with 0.5 g/L PEI and variable PSSA content.

Zeta potential data are shown in Figure 6a. The results for the composite membranes reflect the influence of the particle concentration very well. The membrane prepared only with PEI (equivalent to PSSA content of 0 g/L in Figure 6) had the typical zeta potential with an isoelectric point of pH 9. As the fraction of particles within the coating solution/dispersion increased, the isoelectric point shifted to a more negative value, and the zeta potential became correspondingly more negative. This related well to the single salt rejections and the corresponding water permeabilities. Relevant parts of IR spectra are shown in Figure 6b (complete IR spectra are shown in Figure S3). The band at ~1038 cm^−1^ could be assigned to the symmetric S-O stretching vibration of the sulfonic acids groups as a signature of the PSSA particles. Data clearly reveal that with increasing particle concentration in the coating solution/dispersion, the intensity of the corresponding band also increased.

**Figure 7:**
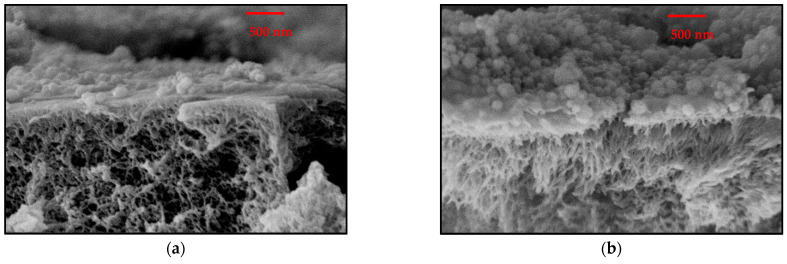
Cross-section SEM images of composite membranes obtained by using different solutions/dispersions for coating: (**a**) 0.25 g/L PSSA particles, 0.5 g/L PEI, and 1 g/L GDE; (**b**) 0.5 g/L PSSA particles, 0.5 g/L PEI, and 1 g/L GDE.

SEM data for selected membranes can be seen in Figure 7. The images show an added layer containing particles on the surface of the PES support membrane in both cases. The distribution of the particles appeared relatively inhomogeneous, but an increase in particle density on the surface can be observed from Figure 7a (0.25 g/L) to Figure 7b (0.5 g/L), which is consistent with the increase in particle fraction used for coating. Particles seem to be partially embedded in a thinner layer; this could be explained by the partial formation of an interpenetrating structure upon mixing both polyelectrolytes that is then cross-linked. It should be considered that images had been taken for the dry membranes; because the cross-linked polyelectrolytes forming the barrier layer will swell in water, the wet structure in operating mode will be different.

Because the barrier layer is formed by the combination of a strong (PSSA particles) and a weak (PEI) polyelectrolyte, the effective charge of the barrier layer will change with the pH value (Figure 6a). Therefore, it can be expected that the rejection pattern for different salts for each specific membrane type (with a certain PSSA:PEI ratio; Figure 5) will also depend on the pH value (analogous to, for example, previous work where Nafion and polyvinylamine had been combined in the barrier layer of a polyelectrolyte complex NF membrane [17]). Because the focus of this work was the demonstration of the feasibility of using PSSA particles as building blocks for the fabrication of tunable polyelectrolyte complex membranes in a simple one-step coating process, this aspect was not further investigated.

In summary, polyelectrolyte barrier layers with structures and properties that are tunable by the particle fraction have been obtained. Up to particle concentrations of 0.25 g/L, the covalent crosslinking between PEI molecules by GDE dominated, and ionic cross-linking between PEI and PSSA (PEC formation) might have an additional contribution. By further increasing the particle concentration up to 1 g/L, the chemical crosslinking between PEI molecules was disturbed, and ionically crosslinked structures PEI and PSSA were predominantly formed. In the apparently charge-balanced barrier layers (according to results of single salt and salt mixture nanofiltration; Figure 5 and Figure S1) obtained at 0.25 g/L, the particles may act as negatively charged domains (with sulfonic acid group) and the PEI (with ammonium groups) as a positively charged matrix. It is believed that the way the two polyelectrolytes are applied to the membrane surface results in the formation of polyelectrolyte complexes, which is also thermodynamically favored [27,28]. However, due to the cross-linking of the PSSA particles (during synthesis) and of the PEI (during membrane fabrication), a complete interpenetration of the two polyelectrolytes is impossible, so the evoked domain structure is plausible.

Unfortunately, none of the membranes prepared here, neither charge-balanced nor with an excess of one charge, showed the rejection pattern expected for CMs in conventional theory [16,29]. Specifically, no membrane showed the rejection of salt of both divalent cation and divalent anions lower than that of monovalent ions. The charge-balanced membranes could fail to yield the expected salt rejection patterns for several reasons. First, it might be because the charged domains were not continuous over the entire barrier layer (e.g., because PEI could fully engulf particles on the PES surface and partially block support membrane pores) or because the particle’s size was too large. However, images in Figure 7 indicate that the PEI matrix and most particles could cross the entire top-layer thickness. It is then likely that the reason might be of a more fundamental nature, as explained below.

In a recent paper, Fan et al. [30] reported that conductivity of an ion-exchange membrane loaded with a counter-ion of different valences was largest for monovalent counter-ions and decreased with valency. This trend was related to the mobilities of counter-ions suppressed by the non-homogeneity of the electric field around fixed charges, predicted within the Manning counter-ion condensation theory, for media of reduced permittivity and increasing with the valency. Essentially, the same conclusion follows from a recently proposed and distinctly different physical picture based on the Bjerrum-like association of fixed charges and counter-ions [31]. Since—as a defining feature of CMs—all ions permeate an ideal CM as counter-ions via their respective domains, the conductivity of domains is directly related to the permeability of the CM. Although partitioning factors—the basis of conventional theory of CMs based on Donnan model—favors multivalent ions, their reduced mobility highlighted by Fan et al. [30] may override the effect of partitioning and preclude the expected CM performance. This scenario obviously needs further investigation and will be clarified in future studies.

An overview of literature data for polyelectrolyte complex membranes made from building blocks with similar ionic groups but using the LBL approach is provided in Table S1 [32,33,34,35,36,37]. The comparison of overall separation performance, i.e., considering the trade-off between water permeance and salt rejection, reveals that the charge-balance membranes, taken as one example, are not yet fully competitive. One option to increase performance due to higher water permeance with same rejection pattern has already been indicated above, i.e., preventing the pore collapse of the support membrane during the thermal curing step.

## 4. Conclusions

In this work, we have reported a novel polyelectrolyte complex NF membrane prepared by a simple one-step coating on a PES ultrafiltration membrane. The negatively charged PSSA particles, as a novel kind of building block, were synthesized by batch emulsion polymerization. Such PSSA particles and positively charged PEI were used for membrane preparation. The membrane charge (zeta potential) and salt rejection could be adjusted by the particle concentration in the coating solution/dispersion that determined the selective layer composition. In this way, membranes with low permeabilities, but balanced charge and the symmetric rejection of divalent ions, could be obtained. The membrane barrier structure and separation performance were controlled by the fraction of particles. At low particle concentrations, the crosslinking of PEI seemed to be more effective. At higher particle concentrations, an increase in permeability could be observed, possibly due to less chemical crosslinking of the PEI and more ionic interaction between PEI and PSSA particles. The membranes obtained at a specific ratio between PSSA and PEI contained domains with either an excess of positive or negative charges, resulting in approximately equal rejection of positively and negatively charged species of higher valency. Overall, the feasibility of synthesizing tailored polyanionic particles and of their integration as building blocks in PEC NF membranes was demonstrated. However, CM behavior was not obtained. The reason might lie in the membrane morphology or be of a more fundamental nature. The domain structure achieved in this work might not meet the stringent requirements that both domains in a CM span the entire thickness and have commensurate permeability controlled by the Donnan mechanism. As an alternative, in the next stage towards an improved structure, positively charged particles may be synthesized and combined with the already established negatively charged particles. On the other hand, the ideal CM performance may also be precluded by the reduced mobility of multivalent ions compared to their monovalent counterparts. Hence, the feasibility and the development of a true CM NF membrane still remain a challenge for future research.

## Figures and Tables

**Table 1:** Size and related polydispersity index, determined by DLS, as well as sulfur content, analyzed by elemental analysis, of the particles after synthesis (“protected”) and after subsequent deprotection. Mean values and standard deviation were calculated from results of three individual measurements.

Sample	Size (nm)	PDI	S (wt%)	Functional/Charged Group Density, Experimental (mmol/g)
**Protected**	207 ± 12	0.14 ± 0.01	14.9 ± 0.1	4.7
**Deprotected**	344 ± 40	0.14 ± 0.06	13.6 ± 0.1	4.3

## Data Availability

Not applicable.

## Supplementary Materials

The following supporting information can be downloaded at https://www.mdpi.com/article/10.3390/membranes12111138/s1, Figure S1. Water permeance and rejection of individual ions in a mixture of three salts for the composite membrane obtained by a coating at a PSSA particle concentration of 0.25 g/L and PEI and GDE concentrations of 0.5 g/L and 1 g/L, respectively. Figure S2. Influence of the variation in PSSA particle content in ethanol used for coating the porous PES support membrane onto water permeance and rejection of single salts. The PEI and GDE contents were 1 g/L (in the second series of experiments). Figure S3. Complete IR spectra of NF membranes from the first series.
