# Supplementary material for: Polystyrene Sulfonate Particles as Building Blocks for Nanofiltration Membranes
Source: Membranes (Basel). 2022 Nov 12;12(11):1138. doi: 10.3390/membranes12111138 (PMC9697654; doi:10.3390/membranes12111138)
Supplement: Supplementary file 1 [file membranes-12-01138-s001.zip › membranes-1967096-supplementary.pdf]

Supporting Information

Polystyrene sulfonate particles as building blocks for nanofiltration membranes

Philipp Jahn[1], Michael Zelner[2], Viatcheslav Freger[2], Mathias Ulbricht[1,*]

[1] Lehrstuhl für Technische Chemie II and Center for Water and Environmental Research (ZWU), Universität Duisburg-Essen, 45117 Essen, Germany
[2] Wolfson Department of Chemical Engineering, Technion - IIT, Haifa, Israel

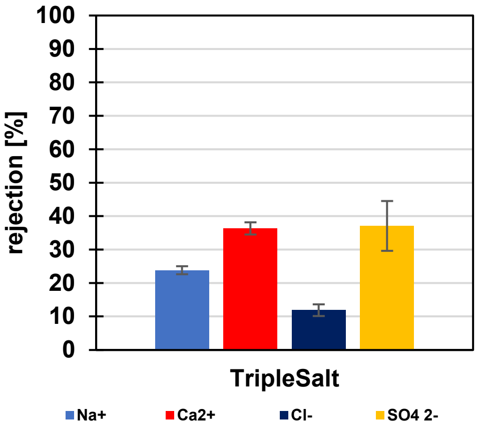

Figure S1. Water permeance and rejection of individual ions in a mixture of three salts for the composite membrane obtained by coating at PSSA particle concentration of 0.25 g/L and PEI and GDE concentrations of 0.5 g/L and 1 g/L, respectively (cf. Figure 5).

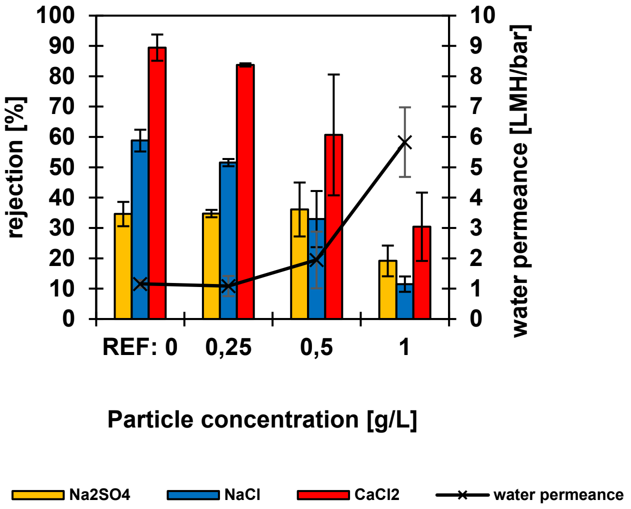

Figure S2. Influence of the variation of PSSA particle content in ethanol used for coating the porous PES support membrane onto water permeance and rejection of single salts. The PEI and GDE contents were 1 g/L (= second series of experiments).

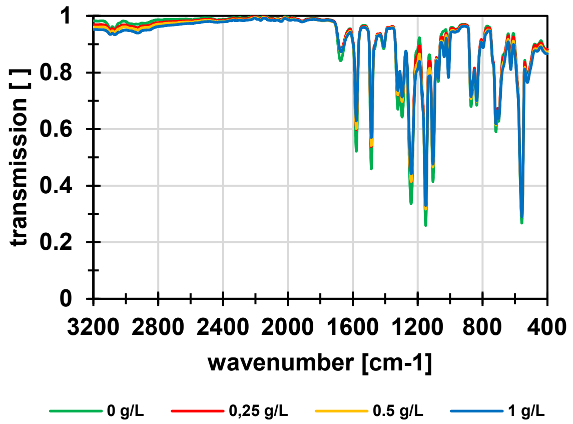

Figure S3. Complete IR spectra of NF membranes from first series.

Table S1. Overview on literature data regarding separation performance for other polyelectrolyte complex nanofiltration membranes in comparison with the charge-balanced membranes obtained in this work.

Membrane[#]	Reference	Water permeance [LMH/bar]	Rejection ($MgSO_4$) [%]	Rejection (Na_2SO_4) [%]	Rejection ($MgCl_2$) [%]	Rejection (NaCl) [%]
dNF40	NX Filtration[1] [32]	5.8	91	-	-	-
dNF80	NX Filtration[1]	-	76	-	-	-
G-CNTm (8:3)	Y. Han et al. [33]	9.5	40.6	83.5	18.7	48.1
GNm	Y. Han et al. [33]	4.8	82.8	95.1	31.7	59
LBL*1.5C	R. Wang et al. [34]	10	97.4	95.2	98.2	33.5
$(PDADAMAC/PSS)_7$ @ 0.05 M NaCl	J. de Grooth et. al. [35]	15.6	-	96	~5[*]	71
$(PDADAMAC/PSS)_7$PDADAMAC @ 0.5 M NaCl	J. de Grooth et. al. [35]	10.3	-	~17	79[*]	~20
$(PSS/PEI)_{10}$ @ 0.05 M NaCl	M. Reurink et. al. [36]	4	~98	~99	~~58	~85
$(PSS/PAH)_{10}$ @ 0.05 M NaCl	M. Reurink et. al. [36]	9	~96	~85	~98	~55
$(PSS/PAS)_{10}$ @ 0.05 M NaCl	M. Reurink et. al. [36]	22	~70	~95	~20	~40
$(PEI/PAA)_{5.5}$ crosslinked	Y. Liu et. al. [37]	~1.4	~80	-	~95	~68
1 g/L PEI; 1 gL PSSA; 1 g/L GDE	this work	1.9		36	61[*]	33
0.5 g/L PEI; 1 gL PSSA; 1 g/L GDE	this work	1		52	50[*]	27

[#] multiwalled carbon nanotubes (MWNT)-intercalated graphene nanofiltration membrane **(GCNTm)**; neat graphene nanofiltration membrane **(GNm)**; poly(diallyldimethylammonium chloride) **PDADAMAC**; poly(styrene sulfonate **(PSS)**; poly(allylamine hydrochloric acid) **(PAH)**; poly(4-aminostyrene) **(PAS)**; polyacrylic acid **(PAA)**, polyethylenimine **(PEI)**, glycerol diglycidyl ether **(GDE)**

[*] $CaCl_2$

[1] Data sheets from NX Filtration

32. D van der Poel, S. Parting ways – removal of salts and organic micropollutants by direct nanofiltration: Pretreatment of surface water for the production of dune infiltration water. **2020**; https://repository.tudelft.nl/islandora/object/uuid%3A6774b91c-6850-4c82-b3c0-a3110f0c40b9
33. Han, Y.; Jiang, Y.; Gao, C. High-Flux Graphene Oxide Nanofiltration Membrane Intercalated by Carbon Nanotubes. *ACS Applied Materials & Interfaces* **2015**, *7*, 8147-8155, doi:10.1021/acsami.5b00986.
34. Liu, C.; Shi, L.; Wang, R. Crosslinked layer-by-layer polyelectrolyte nanofiltration hollow fiber membrane for low-pressure water softening with the presence of SO 4 2− in feed water. *Journal of Membrane Science* **2015**, *486*, 169-176, doi:10.1016/j.memsci.2015.03.050.
35. de Grooth, J.; Oborný, R.; Potreck, J.; Nijmeijer, K.; de Vos, W.M. The role of ionic strength and odd–even effects on the properties of polyelectrolyte multilayer nanofiltration membranes. *Journal of Membrane Science* **2015**, *475*, 311-319, doi:https://doi.org/10.1016/j.memsci.2014.10.044.
36. Reurink, D.M.; Willott, J.D.; Roesink, H.D.W.; De Vos, W.M. Role of Polycation and Cross-Linking in Polyelectrolyte Multilayer Membranes. *ACS Applied Polymer Materials* **2020**, *2*, 5278-5289, doi:10.1021/acsapm.0c00992.
37. Liu, Y.; Chen, G.Q.; Yang, X.; Deng, H. Preparation of Layer-by-Layer Nanofiltration Membranes by Dynamic Deposition and Crosslinking. *Membranes* **2019**, *9*, 20, https://doi.org/10.3390/membranes9020020.
